# The Saudi Population's Preferences: Otolaryngologist vs. Plastic Surgeons for Rhinoplasty

**DOI:** 10.7759/cureus.92846

**Published:** 2025-09-21

**Authors:** Rajab A Alzahrani, Sereen S Aljohani, Maryam Y Almuslem, Fai D Albishri, Abdulrahman A Almaymoni, Kholoud Alsiwed, Ahmed Ali A Khuzayyim, Ali N Jabbari, Ali Almehmadi, Waheeb F Alfadani, Abdulrahman F Kinsara, Wafaa S Taishan

**Affiliations:** 1 Department of Ear, Nose, Throat (ENT), Saudi German Health Group, Jeddah, SAU; 2 Unit of Otolaryngology, Department of Surgery, Faculty of Medicine, Al-Baha University, Al-Baha, SAU; 3 College of Medicine, King Faisal University, Al-Ahsa, SAU; 4 Facility of Medicine, King Faisal University, Al-Ahsa, SAU; 5 Department of Otolaryngology, King Khalid University, Abha, SAU; 6 College of Medicine and Surgery, King Khalid University, Abha, SAU; 7 Department of Otolaryngology - Head and Neck Surgery, King Fahad General Hospital, Jeddah, SAU; 8 Department of Medicine, King Khalid University, Abha, SAU; 9 Department of Otolaryngology, Alhada Armed Forces Hospital, Taif, SAU; 10 Department of Medicine and Surgery, College of Medicine, Umm Al-Qura University, Makkah, SAU; 11 Department of Medicine and Surgery, Umm Al-Qura University, Makkah, SAU; 12 Department of Emergency Medicine, King Faisal Hospital, Makkah, SAU; 13 Faculty of Medicine/Otolaryngology, Al-Baha University, Al-Baha, SAU

**Keywords:** otolaryngologist, plastic surgeons, population, preference, rhinoplasty, saudi arabia

## Abstract

Background: Rhinoplasty is a popular cosmetic surgery worldwide, performed by both otolaryngologists (ENT specialists) and plastic surgeons. In Saudi Arabia, this study aims to explore the population’s preferences regarding the two specialties, factors influencing their decisions, and the role of media in shaping these preferences.

Objectives: Explore Saudi patients’ preferences for otolaryngologists versus plastic surgeons in rhinoplasty, examine perceptions of each specialty’s expertise, assess the impact of factors such as cost and experience on decision-making, and identify the sources of information patients use.

Methodology: This cross-sectional study involved 402 participants from Saudi Arabia, aged 18-80 years, selected using simple random sampling. Data were collected through an anonymous self-administered electronic questionnaire distributed via social media platforms. Data were analyzed using SPSS version 27 (IBM Corp., Armonk, NY), with statistical significance set at *P* < 0.05.

Results: The majority of participants (219, 54.7%) believed ENT surgeons were associated with fewer complications and higher success rates (203, 50.5%) for rhinoplasty. However, 212 (57.2%) respondents preferred plastic surgeons for aesthetic outcomes. The most important decision-making factors were surgeon experience (369, 91.8%), recommendations from medical professionals (312, 77.6%), and cost (221, 55%). Gender and region significantly influenced knowledge scores (*P* = 0.021 and *P* = 0.041, respectively), while marital status was associated with decision-making factors (*P* = 0.035). Media and personal referrals played a substantial role in shaping preferences, with 173 (43%) participants affirming media influence on their selection process.

Conclusions: Preferences for ENT specialists are primarily driven by functional considerations, whereas plastic surgeons are preferred for aesthetic purposes. Gender, region, and marital status play significant roles in shaping preferences. Media and personal recommendations are critical sources of information. Future studies should examine long-term patient satisfaction and how advancements in cosmetic surgery influence population preferences for rhinoplasty surgeons.

## Introduction

Recent advancements have significantly impacted rhinoplasty, the most common cosmetic procedure performed by plastic surgeons and otolaryngologists [1]. This surgery modifies the nose for both functional improvement and aesthetic enhancement [2,3].

Understanding patient desires and concerns is essential for achieving satisfaction, which hinges on cosmetic results and improved breathing [4]. Realistic expectations must be established by comprehending patient motivations and potential outcomes [5].

Understanding a patient's specific aesthetic concerns is crucial for building trust and achieving a positive outcome [6]. Choosing between an otolaryngologist and a plastic surgeon for rhinoplasty can be challenging. This decision requires evaluating their different areas of expertise and approaches to identify the best fit for individual needs [7,8]. Notably, recent years have seen an increase in otolaryngologists performing rhinoplasty within academic settings, potentially impacting the number performed by plastic surgeons [9]. In fact, a 2018 study showed otolaryngologists performing a higher percentage (71%) of rhinoplasties compared to plastic surgeons [10]. Rhinoplasty procedures are on the rise globally, particularly in the Gulf region, with Saudi Arabia being a leader in the number of plastic surgeries performed, with rhinoplasty being the second most popular procedure [11].

While both surgeons possess the necessary skills, there is a lack of comprehensive research on people's preferences for choosing between them for rhinoplasty. This study aims to explore Saudi people's preferences regarding ENT specialists and plastic surgeons in the context of rhinoplasty and explore why people choose between otolaryngologists and plastic surgeons for rhinoplasty.

## Materials and methods

Study design

A cross-sectional survey was conducted from May 1 to August 31, 2024, across the Central, Western, Southern, Eastern, and Northern regions of Saudi Arabia. The study explored Saudi people's preferences for otolaryngologists versus plastic surgeons in rhinoplasty, examined perceptions of each specialty’s expertise, assessed the impact of factors such as cost and experience on decision-making, and identified the sources of information people used. The study also investigated how media portrayals influenced their choice of surgeon for the procedure.

Inclusion and exclusion criteria

We included male and female participants, both Saudi and non-Saudi, residing in Saudi Arabia, aged between 18 and 80 years, who agreed to participate in the survey. Individuals who did not live in Saudi Arabia, were younger than 18 or older than 80 years, or who declined participation were excluded.

Sample size

The estimated sample size, calculated using Cochran's equation, was approximately 385 with a precision level of ±5% and a 95% confidence level. A total of 402 participants were enrolled in the study.

Sampling frame

Data were collected using an anonymous, self-administered, reliable, and validated electronic questionnaire, which was modified to meet the study objectives. The questionnaire was distributed among the general population of Saudi Arabia through social platforms such as WhatsApp and Telegram. The entire participating population was informed in detail about the study aims and data confidentiality. The questionnaire required participants to provide consent before taking part in the study. It was available in two languages, English and Arabic, allowing participants to choose whichever they felt most comfortable with. The questionnaire included sections on sociodemographic data, awareness and knowledge, decision-making factors, sources of information, and preferences regarding otolaryngologists or plastic surgeons for rhinoplasty.

Statistical analysis plan

The study aimed to explore the preferences of the Saudi population regarding ENT and plastic surgeons for rhinoplasty in Saudi Arabia. Data were cleaned using Excel and analyzed with SPSS (version 27, IBM Corp., Armonk, NY). Categorical data were presented as frequencies and percentages, while numerical data were summarized using medians and interquartile ranges. Non-parametric tests were used to assess associations between variables, with statistical significance defined as *P* < 0.05.

Ethical approval 

The study was conducted after obtaining ethical approval on May 29, 2024, from the Institutional Research Board of Al-Baha University (number: REC/SUR/BU-FM/2024/71). The participants were informed about the study aims and assured of data confidentiality, and consent was obtained from each participant before participating in the study.

## Results

The study included 402 participants, the majority of whom were Saudi (*n* = 382, 95%) and female (*n* = 308, 76.6%), with a large proportion aged 18-25 years (*n* = 167, 41.5%). Most participants lived in the Eastern (*n *= 16, 28.9%) and Northern (*n* = 96, 23.9%) regions, and 293 (72.9%) held a bachelor’s degree. Additionally, 158 (39.3%) reported a monthly income of less than 5,000 Saudi Riyals, while only 29 participants (7.2%) reported earning more than 20,000 Saudi Riyals (Table 1).

**Table 1:** Demographic characteristics (n = 402). *n*, number; %, percentage

Variable		*n* (%)
Gender	Male	94 (23.4)
	Female	308 (76.6)
Age (years)	18-25	167 (41.5)
	26-35	86 (21.4)
	36-45	52 (12.9)
	46-55	64 (15.9)
	56 and above	33 (8.2)
Marital status	Single	209 (52.0)
	Married	175 (43.5)
	Divorced	13 (3.2)
	Widowed	5 (1.2)
Nationality	Saudi	382 (95.0)
	Non-Saudi	20 (5.0)
Education	High school or less	74 (18.4)
	Bachelor’s	293 (72.9)
	Master’s	26 (6.5)
	PhD	9 (2.2)
Monthly income (SAR)	<5,000	158 (39.3)
	5,000-10,000	87 (21.6)
	10,000-15,000	69 (17.2)
	15,000-20,000	59 (14.7)
	>20,000	29 (7.2)
Residency region	Central	81 (20.1)
	Northern	18 (4.5)
	Eastern	96 (23.9)
	Western	91 (22.6)
	Southern	116 (28.9)

As shown in Table 2, only 60 participants (14.9%) reported having undergone rhinoplasty, while the majority (*n *= 342, 85.1%) had not. Among those who had undergone surgery, the reported reasons were correction of a birth defect (*n* = 30, 7.5%), enhancement of appearance (*n* = 16, 3.9%), and improvement of breathing (*n* = 14, 3.4%). More than half of those who had not undergone the procedure expressed no intention of having it in the future (*n* = 216, 53.7%). Regarding participants' knowledge of rhinoplasty, more than one-third reported a good to excellent level of understanding of its risks and benefits (*n* = 152, 37.9%), ENT specialists' qualifications (*n *= 129, 32.1%), and plastic surgeons' expertise in the procedure (*n* = 134, 33.3%).

**Table 2:** Participants' knowledge of the rhinoplasty procedure (n = 402). *n*, number; %, percentage

Variable		*n* (%)
Have you had rhinoplasty before?	Yes	60 (14.9)
	No	342 (85.1)
If yes, what was the main reason that prompted you to undergo rhinoplasty?	Enhance appearance	16 (3.9)
	Correct birth defects	30 (7.5)
	Improve breathing/efficiency	14 (3.4)
If no, would you consider having a rhinoplasty in the future?	Yes	69 (17.2)
	No	216 (53.7)
	Unsure	57 (14.2)
Knowledge of the potential risks and benefits of rhinoplasty:	Very poor	46 (11.4)
	Poor	66 (16.4)
	Average	138 (34.3)
	Good	77 (19.2)
	Excellent	75 (18.7)
Familiarity with the qualifications of otolaryngologists (ENT specialists) for rhinoplasty:	Very poor	52 (12.9)
	Poor	90 (22.4)
	Average	131 (32.6)
	Good	58 (14.4)
	Excellent	71 (17.7)
Familiarity with qualifications of plastic surgeons for rhinoplasty:	Very poor	55 (13.7)
	Poor	93 (23.1)
	Average	120 (29.9)
	Good	62 (15.4)
	Excellent	72 (17.9)

**Figure 1:**
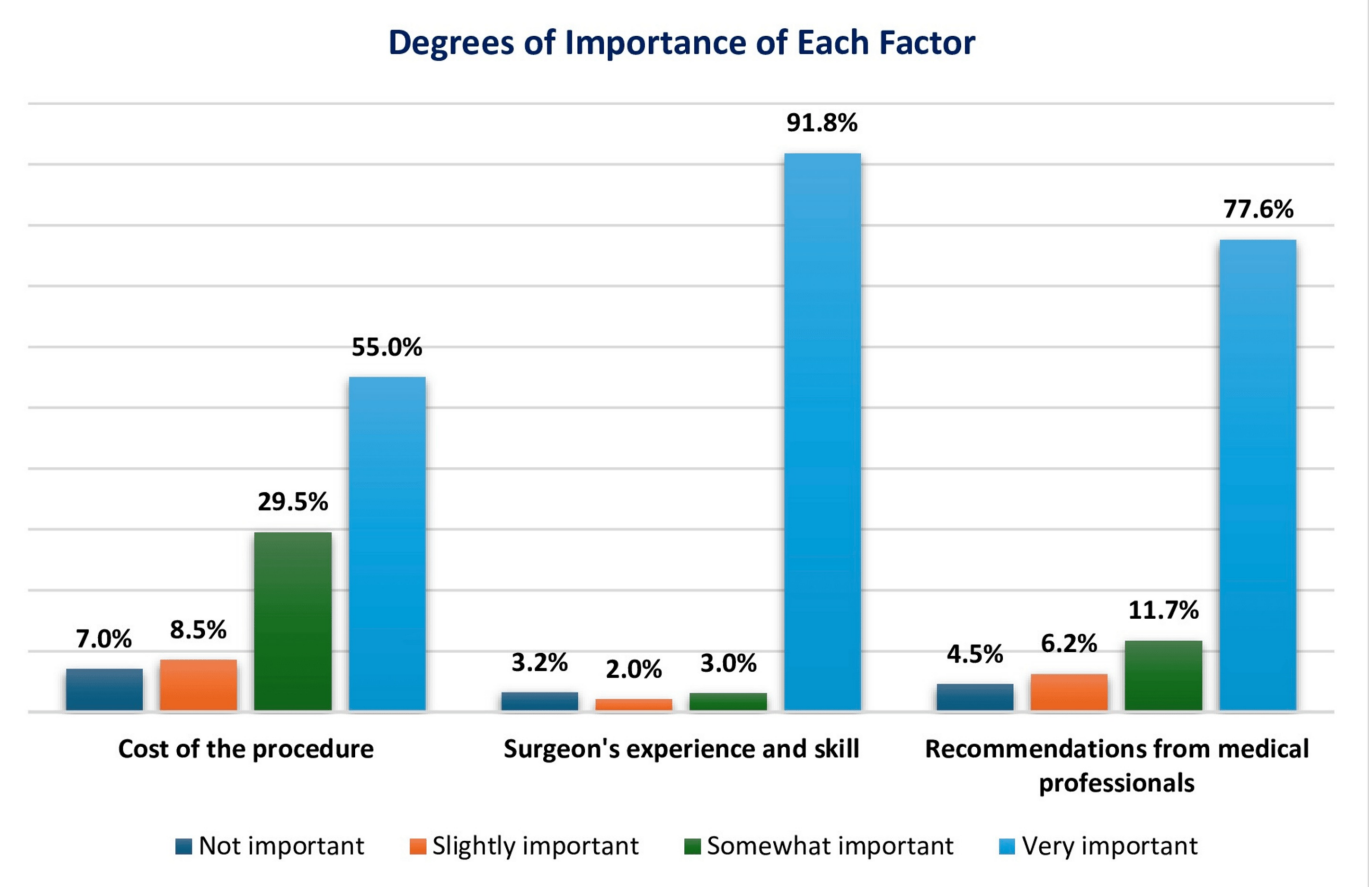
Decision-making factors for rhinoplasty.

Figure 1 illustrates the factors influencing participants' decisions regarding rhinoplasty. The vast majority considered the surgeon's experience and skills (369, 91.8%), recommendations from medical professionals (312, 77.6%), and the cost of the operation as very significant factors while deciding on rhinoplasty.

Most participants in Table 3 obtained information about rhinoplasty and their choice between an otolaryngologist or a plastic surgeon from friends or family (*n* = 231, 57.5%), medical professionals (*n* = 176, 43.8%), social media platforms (*n* = 157, 39.1%), and healthcare websites (*n* = 101, 25.1%). Additionally, a significant number (*n* = 173, 43%) believed that media played a substantial role in shaping their attitude regarding the surgeon they would seek for rhinoplasty, whether an ENT specialist or a plastic surgeon (Table 3).

**Table 3:** Sources of information and media influence on rhinoplasty decision-making. *n*, number; %, percentage

Variable		*n* (%)
Source of information	Personal referrals from friends or family	231 (57.5)
	Recommendations from medical professionals	176 (43.8)
	Social media platforms	157 (39.1)
	Healthcare websites	101 (25.1)
Effect of Media on selecting either ENT or plastic surgeons for rhinoplasty	Not influential at all	41 (10.2)
	Slightly influential	68 (16.9)
	Somewhat influential	120 (29.9)
	Very influential	173 (43.0)

**Figure 2:**
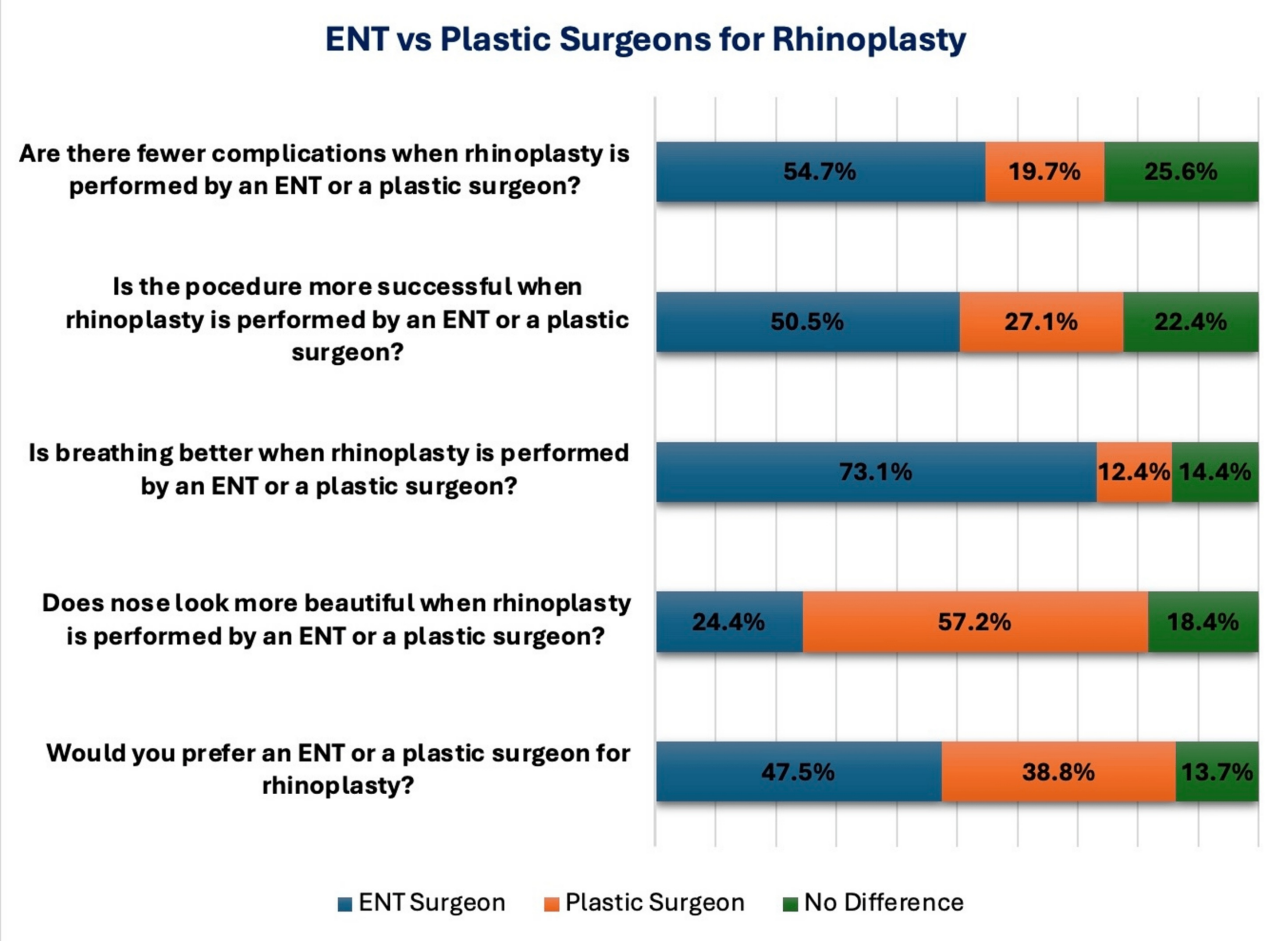
Participants’ preferences for ENT versus plastic surgeons in rhinoplasty.

Participants were also asked about their preference for rhinoplasty performed by either ENT or plastic surgeons. Overall, ENT surgeons were more frequently favored, with the majority (*n* = 219, 54.7%) believing that procedures conducted by ENT specialists carry fewer complications. In line with this, 203 (50.5%) of participants reported higher success rates when an ENT surgeon operates. Thus, a higher proportion (*n* = 191, 47.5%) of participants explicitly chose ENT surgeons for rhinoplasty. Interestingly, while many viewed ENT surgeons as more skilled in the procedure, 212 (57.2%) felt that plastic surgeons were superior in achieving better nose-shaping results, suggesting that aesthetic concerns still play a significant role in influencing surgeon selection (Figure 2).

The total knowledge score was derived from three key questions: knowledge of the potential risks and benefits of rhinoplasty and familiarity with the qualifications of otolaryngologists (ENT specialists) and plastic surgeons for rhinoplasty. Medians for each category were compared to identify significant associations. Overall, knowledge varied significantly based on gender and region of residence. Women demonstrated greater awareness of the procedure (median (interquartile range (IQR)) = 9 (7-12)) than men (*P* = 0.021). Additionally, participants from the Northern region were more knowledgeable about rhinoplasty (median (IQR) = 11 (9-15)) compared to those in the other areas (*P* = 0.041). Similarly, the total score for factors influencing participants' decisions regarding rhinoplasty, including the procedure cost, surgeon experience, and recommendations from medical professionals, was calculated. Marital status was the only variable showing a significant association with decision-making factors, with single participants scoring higher (median (IQR) = 11 (11-12)), indicating that these three factors are particularly important in deciding to undergo rhinoplasty (*P* = 0.035). Other sociodemographic variables did not show a significant association with knowledge or decision-making scores (Table 4).

**Table 4:** The relationship between sociodemographic data and knowledge and decision-making factors. IQR, inter-quartile range

Variable	Category	Knowledge median (IQR)	*P*-value	Test value	Decision-making, median (IQR)	*P*-value	Test value
Gender	Male	9 (6-10)	0.021	*t* = 2.32	11 (9-12)	0.119	*t* = 1.57
	Female	9 (7-12)			11 (11-12)		
Age (years)	18-25	9 (7-12)	0.132	*F* = 1.89	11 (11-12)	0.777	*F* = 0.41
	26-35	9 (6-12)			11 (10-12)		
	36-45	9 (6.25-12)			11.5 (10-12)		
	46-55	9 (7-9)			11 (10-12)		
	56+	9 (7-12)			12 (10-12)		
Marital status	Single	9 (7-12)	0.084	F = 2.11	11 (11-12)	0.035	*F* = 3.04
	Married	9 (6-11)			11 (10-12)		
	Divorced	8 (7.5-14)			11 (9.5-12)		
	Widowed	7 (6-12)			8 (6.5-10.5)		
Nationality	Saudi	9 (7-12)	0.104	*t* = 1.63	11 (10-12)	0.406	*t* = 0.83
	Non-Saudi	7.5 (6-10)			12 (10.25-12)		
Education	High school or less	9 (6.75-11)	0.078	*F* = 2.27	11 (10-12)	0.454	*F* = 0.91
	Bachelor's	9 (7-12)			11 (11-12)		
	Master's	8 (6-11)			11 (9-12)		
	PhD	11 (9-14)			11 (10-12)		
Monthly income (SAR)	<5,000	9 (7-11)	0.236	*F* = 1.42	11 (11-12)	0.862	*F* = 0.23
	5,000-10,000	10 (7-13)			11 (10-12)		
	10,000-15,000	9 (6-11.5)			11 (9.5-12)		
	15,000-20,000	9 (7-12)			11 (11-12)		
	>20,000	9 (7-12.5)			11 (10-12)		
Residency region	Central	9 (6.5-12)	0.041	*F* = 2.51	11 (10-12)	0.662	*F* = 0.67
	Northern	12 (9-15)			11 (9.75-12)		
	Eastern	9 (7-11)			11 (11-12)		
	Western	9 (7-12)			11 (11-12)		
	Southern	9 (7-11)			12 (10-12)		

## Discussion

Rhinoplasty is one of the most common cosmetic surgeries globally, with various reasons for undergoing the procedure, ranging from medical corrections to aesthetic enhancements [12]. In Saudi Arabia, the choice between otolaryngologists (ENT specialists) and plastic surgeons for rhinoplasty has become a topic of growing interest. This study aims to explore the preferences and knowledge of the Saudi population regarding rhinoplasty, with a specific focus on their understanding of the qualifications required for otolaryngologists and plastic surgeons to perform this operation. The demographic profile in our study revealed a predominance of young, female participants, with a large majority holding a bachelor's degree, which may reflect a higher level of education and awareness about cosmetic procedures. This aligns with findings from Morait et al., who noted that cosmetic procedures in Saudi Arabia are more prevalent among younger, educated demographics, particularly women, and tend to be concentrated in urban areas with better healthcare facilities [13]. Income distribution in the sample indicates that (*n* = 158, 39.3%) of participants earn less than 5,000 Saudi Riyals per month, while only a small fraction (*n* = 29, 7.2%) earns over 20,000 Saudi Riyals. In comparison to global data, income plays a significant role in the affordability and accessibility of cosmetic procedures. A study emphasizes that higher income groups are more likely to pursue aesthetic surgery due to greater disposable income [14].

In the present study, only (*n *= 60, 14.9%) of participants reported undergoing rhinoplasty, a figure comparable to similar studies conducted in the region. For example, a study by Alsubeeh et al. reported that around 10% to 15% of surveyed individuals in a cosmetic surgery population in the Middle East had undergone rhinoplasty [15]. The most common reason for rhinoplasty in our sample was correcting a birth defect (*n *= 30, 7.5%), followed by enhancing appearance (*n *= 16, 3.9%) and improving breathing efficiency (*n *= 14, 3.4%). This differs slightly from other studies, where aesthetic reasons are often the leading motivator for rhinoplasty. A study by Amiri et al. found that aesthetic reasons (such as enhancing appearance) were the primary motivators for rhinoplasty in over 60% of cases in the UAE [16]. This finding is also in line with the findings of a study, which reported that cultural perceptions significantly influence the decision to undergo cosmetic surgery [17]. Another notable aspect is that (*n* = 216, 53.7%) of participants who have not undergone rhinoplasty expressed no interest in considering the procedure in the future. This is consistent with a study, which found that individuals in regions with more conservative cultural norms, such as the Middle East, are often hesitant to undergo elective cosmetic surgeries [16]. Knowledge of rhinoplasty risks and benefits among participants was generally average to good, with 138 (34.3%) reporting an average understanding and 152 (37.9%) indicating good to excellent knowledge. Similar trends were found in studies on public awareness of cosmetic surgery, where educational campaigns and increasing exposure to media have been shown to improve general awareness [18]. However, our sample showed a significant portion with poor or very poor knowledge (*n* = 111, 27.8%), indicating that there may still be a gap in public understanding, particularly in regions where cosmetic procedures are less common [19]. Regarding the qualifications of otolaryngologists and plastic surgeons for rhinoplasty, participants reported similar levels of familiarity, with around 32% being familiar with both specialists' qualifications. Studies like that of Mandavia et al. suggest that in the Middle East, the choice of surgeon may often be influenced by the perceived expertise in specific types of surgeries, with otolaryngologists preferred for functional corrections and plastic surgeons for aesthetic improvements [20].

According to the data, surgeons’ experience and skill emerged as the most important factor, with (*n *= 369, 91.8%) of respondents considering it *very important*. This is consistent with previous studies, such as those by Blasberg et al., showing that patients’ trust in the surgeon's competence significantly impacts their decision to proceed with surgery, particularly in complex procedures like rhinoplasty [21]. The second most important factor was recommendations from medical professionals, with 312 (77.6%) of participants rating it as *very important*. This finding is in line with research by Pearlman et al., which found that word-of-mouth recommendations and advice from trusted healthcare providers are influential in elective surgery decisions [22]. Regarding the cost of the procedure, 221 (55%) participants viewed it as a *somewhat important* factor, while only 28 (7%) deemed it *not important*. This finding aligns with global trends, where cost is considered a secondary concern after safety and quality. A study by Hassell et al. found that while price is a factor, patients are more willing to invest in procedures that ensure favorable outcomes, particularly in rhinoplasty, which is often viewed as a long-term investment in one’s appearance [23].

Regarding the source of information, our study found that 231 (57.5%) participants rely on personal referrals from friends or family for decisions about rhinoplasty. Elboraei et al. also highlight that in Saudi Arabia, personal networks strongly influence cosmetic surgery choices, as individuals trust the experiences of friends and family over anonymous sources [24]. Recommendations from medical professionals influence 176 (43.8%) respondents, reinforcing the trust placed in healthcare providers. This aligns with the observed trend that expert opinions are crucial in guiding decisions about specialized medical procedures, as seen in the broader context of elective surgeries where patients often rely on professional guidance to navigate their options [22]. Social media platforms also played a notable role, influencing 157 (39.1%) participants. The rise of social media has had a significant impact on the field of cosmetic surgery globally, and our findings mirror international trends. Studies by Obeid et al. found that platforms like Instagram and Snapchat increase the visibility of cosmetic procedures and shape patient expectations [25]. Healthcare websites influenced 101 participants (25.1%), which is consistent with the findings of Husain et al., who noted the growing role of online resources [26]. Media influence was strong, with 173 (43%) participants considering it very influential in choosing between an ENT specialist or plastic surgeon. Walker et al. noted that media portrayals of cosmetic procedures influence public attitudes and may skew preferences toward more visible specialists [27].

In our study, most participants believed that ENT surgeons were associated with fewer complications (*n* = 219, 54.7%) and higher success rates (*n* = 203, 50.5%) in rhinoplasty. This perception is supported by Hanege et al., who note ENT surgeons' expertise in managing functional aspects and minimizing complications [4]. However, 212 (57.2%) respondents felt that plastic surgeons achieved better aesthetic results, aligning with a study, which highlights plastic surgeons' skills in achieving superior cosmetic outcomes [28]. Additionally, 293 (73.1%) participants believed breathing was better when rhinoplasty was performed by an ENT surgeon. When choosing between surgeons, participants favored ENT specialists (*n* = 191, 47.5%) over plastic surgeons (*n* = 156, 38.8%), likely reflecting a prioritization of function and reduced risk [29].

The results show that gender had a significant association with the total knowledge score, where women exhibited greater awareness (median (IQR) = 9 (7-12)) than men (*P* = 0.021). This finding is consistent with research, which suggests that women tend to have higher levels of interest and engagement in cosmetic procedures, thereby seeking more information about the risks, benefits, and surgeons’ qualifications. Women are also more likely to follow media and social discussions surrounding beauty and aesthetics, contributing to their greater knowledge [13]. In terms of decision-making factors, marital status was the only variable that showed a significant association. Single participants (median (IQR) = 11 (11-12)) scored higher in terms of the importance they placed on factors like procedure cost, surgeon experience, and medical recommendations (*P *= 0.035). This finding is in line with other research, such as Amiri et al., who indicate that single individuals, particularly younger adults, are more likely to consider cosmetic procedures for self-enhancement, and therefore, they tend to weigh decision-making factors more carefully [16]. Interestingly, other sociodemographic factors, such as age, education level, and monthly income, did not show significant associations with knowledge or decision-making factors in this study. These findings contrast with some international research, where higher education and income levels are often linked to greater awareness and a higher likelihood of pursuing cosmetic surgery [30-32]. This lack of significant association could reflect cultural differences in Saudi Arabia, where factors like family influence and societal norms may play a more prominent role in shaping individuals’ decisions about cosmetic procedures than personal financial or educational background [17].

Limitations

This study has several limitations. First, the sample size, though adequate, may not fully represent the broader Saudi population, limiting generalizability. Second, the use of self-reported data may introduce bias, as participants might not recall or understand medical details accurately. Third, data collection through online platforms might exclude less tech-savvy individuals, potentially skewing results. Additionally, this study focused on a specific demographic, and preferences may vary in other regions or cultures. Lastly, the study did not explore long-term satisfaction post-surgery, limiting insights into outcomes over time.

## Conclusions

The preference for otolaryngologists over plastic surgeons for rhinoplasty in Saudi Arabia appears to be driven by the belief that ENT specialists are better suited to address functional concerns, such as improving breathing and reducing complications. However, plastic surgeons remain preferred for aesthetic outcomes, with many participants viewing them as more skilled in nose shaping. Gender and region played significant roles in knowledge levels, while marital status influenced decision-making factors. Personal recommendations, surgeon experience, and medical advice were key in shaping preferences. The media also played a substantial role, highlighting the need for accurate portrayals of both specialties. Future research should explore long-term patient satisfaction and examine how evolving medical advancements impact surgeon preference in cosmetic surgery.
